# Real-Time Endoscopic Video Enhancement via Degradation Representation Estimation and Propagation

**DOI:** 10.3390/jimaging12030134

**Published:** 2026-03-16

**Authors:** Handing Xu, Zhenguo Nie, Tairan Peng, Xin-Jun Liu

**Affiliations:** 1 Department of Mechanical Engineering, Tsinghua University, Beijing 100084, China; xhd21@mails.tsinghua.edu.cn (H.X.);; 2 Beijing Key Laboratory of Transformative High-End Manufacturing Equipment and Technology, Tsinghua University, Beijing 100084, China; 3 State Key Laboratory of Tribology in Advanced Equipment, Tsinghua University, Beijing 100084, China

**Keywords:** real-time video enhancement, representation propagation, image degradation, endoscopic video

## Abstract

Endoscopic images are often degraded by uneven illumination, motion blur, and tissue occlusion, which obscure critical anatomical details and complicate surgical manipulation. This issue is particularly pronounced in single-port endoscopic surgery, where the imaging capability of the camera is further constrained by limited working space. While deep learning-based enhancement methods have demonstrated impressive performance, most existing approaches remain too computationally demanding for real-time surgical use. To address this challenge, we propose an efficient stepwise endoscopic image enhancement framework that introduces an implicit degradation representation as an intermediate feature to guide the enhancement module toward high-quality results. The framework further exploits the temporal continuity of endoscopic videos, based on the assumption that image degradation evolves smoothly over short time intervals. Accordingly, high-quality degradation representations are estimated only on key frames at fixed intervals, while the representations for the remaining frames are obtained through fast inter-frame propagation, thereby significantly improving computational efficiency while maintaining enhancement quality. Experimental results demonstrate that our method achieves an excellent balance between enhancement quality and computational efficiency. Further evaluation on the downstream segmentation task suggests that our method substantially enhances the understanding of the surgical scene, validating that implicitly learning and degradation representation propagation offer a practical pathway for real-time clinical application.

## 1. Introduction

Minimally invasive surgery (MIS) has become a cornerstone of modern clinical practice, offering reduced surgical trauma, shorter recovery times, and improved postoperative outcomes compared with traditional open procedures [1]. Among various MIS techniques, endoscopic surgery plays a particularly critical role, as it enables surgeons to access deep or delicate anatomical regions through narrow working channels with minimal disruption of surrounding tissues [2]. For example, in spine surgery, endoscopic approaches have been increasingly adopted for the treatment of conditions such as lumbar disc herniation, spinal stenosis, and degenerative diseases [3]. Unlike open surgical procedures, MIS relies entirely on intraoperative endoscopic video as the sole source of visual feedback, making video quality a decisive factor for surgical safety and outcome.

However, endoscopic imaging is inherently constrained by complex in vivo conditions, which give rise to various forms of image degradation. The near-axis illumination of cold light sources often results in pronounced spatial non-uniformity in lighting, while strong surface reflectance and light scattering induced by intraoperative fluids further reduce both global and local image contrast. Further, blood contamination, surgical smoke, and instruments frequently enter the field of view, causing persistent dynamic occlusions, whereas continuous endoscope motion within confined spaces and a limited depth of field introduce motion blur and defocus. These degradation factors commonly coexist with temporal and spatial variability, leading to complex and non-stationary degradation patterns in endoscopic videos. Such compounded degradations substantially compromise the visibility of fine anatomical structures, hinder accurate surgical manipulation, and may increase the risk of complications.

To address these challenges, video enhancement techniques have been widely investigated. Conventional approaches [4,5,6,7] can provide modest improvements in brightness or contrast, but they often fail under severe degradations and may amplify noise or introduce artifacts [8]. More recently, deep learning-based methods have demonstrated remarkable success in image restoration tasks, including denoising, deblurring [9], and super-resolution [10,11], by leveraging large-scale data and learning complex degradation models. These techniques have been gradually extended to the domain of endoscopic imaging, with promising results in improving surgical visibility.

Nevertheless, several critical limitations remain. Most existing methods are designed and evaluated in offline settings, where computational efficiency is not a primary concern. As a result, they often involve complex models or iterative optimization procedures that are computationally expensive and unsuitable for deployment in the operating room. In real surgical environments, enhancement must be performed at video frame rates to provide surgeons with immediate visual feedback, typically requiring real-time processing at full HD (1080p) resolution and 25–30 fps with an end-to-end latency below 100 ms. The inability of current methods to achieve real-time restoration, therefore, represents a substantial barrier to clinical adoption.

The complex and dynamically evolving degradations in endoscopic images exhibit substantial temporal and spatial variability, making it challenging to achieve high-quality enhancement using a single end-to-end deep learning model without resorting to large and computationally expensive architectures. To overcome this limitation, we propose a stepwise enhancement framework that explicitly decouples degradation estimation from image enhancement. Specifically, an implicit degradation representation is introduced to compactly characterize the overall degradation state of each frame and to guide the enhancement process. Under the assumption that image degradation varies smoothly over short temporal intervals, high-quality degradation representations are estimated only on sparsely sampled key frames and stored in a degradation memory bank. For intermediate frames, the corresponding degradation representations are efficiently obtained through fast propagation from the memory bank, enabling substantial improvements in inference efficiency while maintaining enhancement quality. Qualitative and quantitative comparisons with other video or image enhancement methods demonstrate the superior performance and efficiency of the proposed method in enhancing endoscopic videos. Further evaluation on the downstream segmentation task suggests that our method substantially enhances the understanding of the surgical scene. A preliminary version of this work has been released as a preprint on arXiv [12].

## 2. Related Work

### 2.1. Endoscopic Video Enhancement

The enhancement of endoscopic video streams is a critical research area focused on correcting dynamic degradations to provide a clear and stable view for clinicians. Algorithmic solutions primarily target motion blur and various visual artifacts that compromise video quality. Early methods that processed each frame independently were often insufficient, as they failed to account for the temporal nature of video, leading to inconsistencies and suboptimal results. Consequently, modern research heavily utilizes the temporal context available in video sequences. For instance, in addressing motion blur caused by physiological movement or instrument instability, synthesis-based methods have proven effective. These techniques restore sharpness in blurred frames by identifying and transferring high-frequency details from adjacent clear frames, a process demonstrated by [13] using a non-parametric motion model to align the frames accurately.

The advent of deep learning has also marked a paradigm shift in handling image artifacts. Of the various artifacts that degrade endoscopic images, specular reflections are particularly problematic, as they can saturate parts of the image and obscure critical underlying tissue. While traditional methods relied on filtering and inpainting, deep learning models can now intelligently detect these highlights and reconstruct the missing information. Beyond reflections, these models have excelled at removing other common obstructions. The field has evolved from physics-based models, such as the chromaticity-based approach for smoke removal proposed by [14], to more advanced deep learning solutions like improved U-Net architectures that perform real-time desmoking [15]. Furthermore, to combat the inherent noise in endoscopic sensors, CNN-based blind denoising methods have been developed to handle noise without requiring prior information about its statistical properties [16,17]. Other specialized models effectively eliminate bubble artifacts in wireless capsule endoscopy videos, which frequently obstruct the view of the gastrointestinal tract [18].

### 2.2. Real-Time Image Enhancement

Real-time image enhancement provides immediate visual improvement during live procedures, a crucial capability for on-the-fly diagnosis and intervention. This domain is now dominated by deep learning models that offer both the rapid processing speed and the sophisticated analytical power required for clinical applications. Super-resolution (SR) is a vital technique for improving detail from low-resolution sources, such as capsule endoscopes, or for reducing bandwidth requirements in video streaming. The field has progressed rapidly from foundational CNNs to advanced architectures. Modern models like EndoL2H [19] use Generative Adversarial Networks (GANs) and spatial attention mechanisms to generate high-fidelity, photorealistic images. The latest trend involves hybrid models that combine CNNs with transformers to capture both local textural features and long-range contextual dependencies, as seen in HA-VSR [20] and E-SEVSR [21]. Additionally, saliency-aware and attention mechanisms are being integrated to focus computational resources on diagnostically relevant regions, further boosting performance and efficiency [22,23].

For low-light conditions that can obscure critical anatomical features, Retinex-based deep networks like EIEN [24] have been developed to correct illumination while preserving natural colors and details. Unsupervised models such as EnlightenGAN [25] offer greater flexibility by not requiring paired training data, which is often difficult to acquire in medical settings. Beyond improving raw image quality, deep learning powers real-time computer-aided detection (CADe) and diagnosis (CADx) systems by highlighting suspicious areas for clinicians as a “second observer”. CNN models can detect and classify pathologies like polyps [26] and early-stage cancers [27] in video streams at speeds exceeding 25 frames per second, well within the requirements for real-time clinical use. Recent advancements include multi-task models like DSI-Net [28] for joint classification and segmentation and robust ensemble learning techniques that improve accuracy and reliability by combining predictions from multiple models [29,30].

## 3. Methodology

### 3.1. Video Enhancement Framework

The proposed endoscopic video enhancement framework is illustrated in Figure 1. In this framework, each frame of the input video sequence undergoes degradation representation modeling and enhancement generation. Specifically, for frames whose index is a multiple of $T_{\Delta }$, the system invokes the degradation-aware module to perform a full estimation, yielding high-precision degradation features. For frames that are not multiples of $T_{\Delta }$, the degradation representation is rapidly estimated through the DRPM along the temporal dimension and subsequently fed into the generator to enhance the current frame.

This “key-propagation” design enables the framework to maintain accurate degradation representations without performing high-complexity modeling on every frame, thereby significantly reducing the overall computational load. More importantly, the propagation mechanism ensures strong temporal consistency and continuity across the video sequence, allowing the system to produce stable enhancement results while satisfying real-time requirements.

### 3.2. Single Frame Enhancement Model

We introduce a single-frame image enhancement model that is guided by degradation representation. Building upon the CycleGAN framework [31], our approach leverages cyclical consistency between degraded and restored images to construct the training paradigm. Within the generator, we design a dedicated degradation-aware module (DAM) to extract the implicit degradation characteristics of the input image, which are then used to guide the enhancement of the current frame. This design ensures that the enhancement process is explicitly aware of image degradation, leading to more reliable restoration in complex surgical scenarios.

#### 3.2.1. Degradation-Aware Module

This module learns to characterize degradation representations through a contrastive learning strategy inspired by MoCo [32,33]. As illustrated in Figure 2a, given a batch of random degraded images *X*, the assumption is that only representations extracted from the same image should be identical, while those from different images should remain distinct. Given an image patch as the query patch, other patches from the same image can be considered as positive samples. In contrast, patches from other images are referred to as negative samples. To enforce this property, the degradation-aware module is trained using the InfoNCE loss [34] as (1), which measures the similarity among multiple degradation representations.

(1) $$L_{\mathrm{con}}=-\log\frac{\exp(d_{i}^{q}\cdot d_{i}^{k}/\tau )}{\sum_{j=1}^{N}\exp\left(d_{i}^{q}\cdot d_{j}^{k}/\tau \right)}$$

To strengthen the expressive capacity of the DAM, we replace conventional stacked convolutional layers with a series of residual blocks, as shown in Figure 2b. All other settings follow the configuration described in [33].

The extracted degradation representation *d* is high-dimensional and often redundant. To obtain a compact yet informative embedding, we introduce both channel attention and spatial attention mechanisms as (2), which selectively emphasize the most relevant degradation cues while suppressing irrelevant information (Figure 3b).

(2) $$\begin{array}{c} d^{\prime }=\sigma \left(W_{c2}\delta \left(W_{c1}d\right)\right)*d\\ d_{c}=\sigma \left(W_{s1}d^{\prime }\right)*d^{\prime } \end{array}$$

where σ,δ represent the sigmoid and ReLU activation function separately.

This architectural refinement allows the encoder to capture richer and more discriminative degradation features. Importantly, by producing a more accurate and compact representation of degradation, the DAM reduces the burden on the subsequent image enhancement module. As a result, the overall framework not only improves the quality of degradation modeling but also lowers the complexity of the enhancement stage, which is essential for achieving real-time endoscopic video enhancement.

#### 3.2.2. Degradation Guided Enhancement Module

As shown in Figure 3a, building on the degradation representation generated by the DAM, we design a degradation guided enhancement module (DGEM) that generates the enhanced image in three sequential stages.

Shallow feature extraction. We extract low-level structural information from the input image using two stacked 3×3 convolutional layers. This step preserves local edges and textures, which are essential for fine-grained restoration.

Feature modulation. Inspired by the Swin Transformer [35], we adopt a shifted-window attention mechanism to capture both local and long-range dependencies. Building upon the standard multi-head attention mechanism, we introduce a degradation-aware modulation applied specifically to the value vectors in each attention head, as shown in Figure 3c. As shown in (3), by incorporating the degradation representation into the values, the attention module can adaptively emphasize or suppress features according to the estimated degradation in the input image.

(3) $$\begin{array}{c} \hat{V}=V⊙d\\ \mathrm{MSA}\left(I\right)=\left[{\mathrm{Concat}}_{h=1}^{H}\left(\mathrm{Softmax}\left(\frac{Q_{h}K_{h}^{T}}{\sqrt{d_{h}}}\right)\hat{V}\right)\right]W_{o} \end{array}$$

where *d* denotes the compressed degradation representation, $Q_{h}$, $K_{h}$, and $V_{h}$ correspond to the query, key, and value of the h_th attention head, and $d_{h}$ represents the dimensionality of each head.

Unlike modulating the query or key vectors, which would alter the attention distribution itself, modulating the Values directly adjusts the content carried through the attention mechanism, allowing the network to integrate degradation information without disrupting spatial relationships. This strategy enables more effective feature aggregation that is guided by the current degradation conditions, ultimately improving the fidelity and robustness of the enhanced image.

Reconstruction. Finally, the modulated features are passed into a sub-pixel convolution upsampling module, which ensures that the final output benefits from degradation-aware guidance while maintaining high spatial fidelity.

#### 3.2.3. Cyclical Consistency

In the CycleGAN framework, style transfer between two image domains is achieved by employing two generators and two discriminators under the constraint of cyclical consistency. Inspired by this idea, we construct a cyclical consistent adversarial network between the low-quality and high-quality image domains, denoted as L,H, respectively.

As illustrated in Figure 4a, the enhancement process L→H is carried out by the combination of DAM and DGEM, while the degradation process H→L is directly implemented by predefined degradation models (PDMs) based on degradation parameters $d_{p}$. For simplicity, we use $G_{H}$ to denote the combination of DAM, DGEM and use $G_{L}$ to denote PDMs, respectively. Meanwhile, the appearance discriminators $D_{L},D_{H}$ compete with the corresponding generators to correctly differentiate the synthetic images from the real images, denoted by (4) and (5). To encourage the generation of high-quality enhanced images, we extend the standard two-discriminator framework by adding a high-frequency discriminator $D_{hf}$ that focuses specifically on the high-frequency of the high-quality image domain. This design aims to guide the generator in preserving fine-grained details and texture information, and its corresponding adversarial loss is defined as (6).

(4) $$\begin{array}{c} \begin{array}{cc} L_{\mathrm{adv}}\left(G_{H},D_{H}\right)= & E_{x_{h}∼p\left(x_{h}\right)}\left[\log\left(D_{H}\left(x_{h}\right)\right)\right]+\\ & E_{x_{l}∼p\left(x_{l}\right)}\left[\log\left(1-D_{H}\left(G_{H}\left(x_{l}\right)\right)\right)\right] \end{array} \end{array}$$

(5) $$\begin{array}{c} \begin{array}{cc} L_{\mathrm{adv}}\left(G_{L},D_{L}\right)= & E_{x_{l}∼p\left(x_{l}\right)}\left[\log\left(D_{L}\left(x_{l}\right)\right)\right]+\\ & E_{x_{h}∼p\left(x_{h}\right)}\left[\log\left(1-D_{L}\left(G_{L}\left(x_{h}\right)\right)\right)\right] \end{array} \end{array}$$

(6) $$\begin{array}{c} \begin{array}{cc} L_{\mathrm{adv}}\left(G_{H},D_{hf}\right)= & E_{x_{h}∼p\left(x_{h}\right)}\left[\log\left(D_{hf}\left(F_{hp}\left(x_{h}\right)\right)\right)\right]+\\ & E_{x_{l}∼p\left(x_{l}\right)}\left[\log\left(1-D_{hf}\left(F_{hp}\left(G_{H}\left(x_{l}\right)\right)\right)\right)\right] \end{array} \end{array}$$

where $F_{hp}$ denotes a highpass filter.

It should be note that, shown as the purple part of Figure 4a, different multilayer perceptrons (MLPs) are utilized to regress degradation parameters involved in different image degradation models. These MLPs estimate explicit degradation parameters $d_{p}$ from the learned implicit degradation representation $d_{c}$, which are then used to synthesize degraded images back. Taking the classical image blurring model $\hat{I}=\sigma ⊗I+n$ as an example, the regression module can estimate a spatially varying blur kernel ${\hat{\sigma }}_{i,j}$ together with a noise intensity field ${\hat{n}}_{i,j}$. These estimated parameters are further used to degrade the enhanced images back to the low-quality domain L, thereby enabling training with paired high- and low-quality images. Importantly, by grounding the degradation process in physically meaningful models rather than in an unconstrained learned generator, our framework avoids introducing unrealistic artifacts and ensures that the cyclical consistency better reflects the actual imaging degradation.

As shown in Figure 4b, the cyclical consistency loss is defined as (7), which forces the reconstructed synthetic images $G_{L}\left(G_{H}\left(l^{A}\right)\right)$ and $G_{H}\left(G_{L}\left(h^{B}\right)\right)$ to be identical to their respective inputs $l^{A}$ and $h^{B}$.

(7) $$\begin{array}{cc} L_{\mathrm{cyc}}\left(G_{H},D\right)= & \;E_{l^{A}∼p\left(A\right)}\left[\right\|G_{L}\left(G_{H}\left(l^{A}\right)\right)-l^{A}{\|}_{1}]+\\ & \;E_{h^{B}∼p\left(B\right)}\left[\right\|G_{H}\left(G_{L}\left(h^{B},d_{c}^{A}\right)\right)-h^{B}{\|}_{1}]+\\ & \;E_{l^{A},h^{B}∼p\left(A,B\right)}[\|D\left(G_{L}\left(h^{B},d_{c}^{A}\right)\right)-D\left(l^{A}\right){\|}_{1}] \end{array}$$

where the third term denotes the cyclical consistency of the degradation representation, and *D* in it represents the DAM. It should be mentioned that the degradation representation $d_{c}$ can be only produced by the DAM within $G_{H}$. Consequently, in the term $G_{L}\left(h^{B},d_{c}^{B}\right)$, the degradation representation $d_{c}^{B}$ can only be replaced with $d_{c}^{A}$ that derived from $G_{H}\left(l^{A}\right)$, which means that the degradation representation cycle contains only a single term.

### 3.3. Degradation Representation Propagation Module

Although the aforementioned single-frame image enhancement model can achieve high-quality endoscopic image restoration, its inference efficiency is insufficient to meet the real-time requirements of intraoperative observation. To address this, we balance the complexity of different model components against overall performance. Specifically, we manually adjust the number of residual blocks and Swin Transformer blocks to adjust the number of parameters and floating-point operations (FLOPs) for each module (detailed in Table 1), concentrating the primary computational cost in the degradation representation extraction stage.

Based on these adjustments, we further introduce a degradation representation propagation module (DRPM) to fully exploit temporal correlations across video frames. This module leverages the degradation representations of previous frames as implicit priors, propagating and updating them along the temporal dimension to efficiently estimate the degradation representations of future frames. Compared with modeling each frame independently, this propagation mechanism significantly reduces redundant computations by avoiding repeated processing of highly similar information across adjacent frames, thereby lowering overall computational complexity and ensuring real-time enhancement. Moreover, by maintaining inter-frame consistency of the degradation representations, the module enhances the stability and temporal coherence of degradation modeling across sequences.

In implementation, the DRPM is built upon a Transformer architecture as shown in Figure 5. Unlike conventional recurrent neural networks (RNNs) or 3D convolutional networks (3D-CNNs), the self-attention mechanism in Transformers enables flexible modeling of dependencies over a long temporal range, overcoming the locality constraints of convolution or the limited temporal span of RNNs. Through multi-head attention, the module can simultaneously capture multi-scale temporal correlations in the degradation representations, accurately modeling both local dynamics between adjacent frames and long-range dependencies across distant frames. As a result, employing a Transformer not only improves the accuracy and robustness of degradation representation estimation but also preserves temporal consistency and overall stability in the enhanced video sequence.

Importantly, to further improve the efficiency of degradation propagation while maintaining effectiveness, the module does not propagate the full degradation representation *D* directly. Instead, it uses a compressed representation $\hat{D}$ as the temporal modeling unit, which is not directly illustrated in Figure 1. This strategy reduces feature dimensionality and computational redundancy while also enhancing the model’s generalization and real-time performance, providing an efficient degradation-aware support for continuous-frame endoscopic image enhancement.

### 3.4. Training Strategy

The training strategy is detailed in Algorithm 1. In the first $N_{d}$ epochs, the model first pre-trains the DAM using the contrastive loss $L_{con}$ on paired endoscopic images generated by artificial degradations, enabling it to capture effective degradation representations. After this stage, we switch to real-world data, training the model on unpaired low-quality and high-quality endoscopic images under the CycleGAN paradigm. During adversarial learning, to train the generators, the associated objective is minimized, while to train the discriminators, the associated objective is maximized. Please note that when the generators are optimized, the weights of the discriminators are fixed and vice versa. At each step, we conduct the following computations, including forward propagation, loss calculation, backward propagation, and updating weights of associated components. Finally, for video datasets, non-key frames are processed by replacing the DAM in the single-frame enhancement model with the DRPM. The training is then conducted using the same loss functions as described above, which guide the DRPM to propagate and generate reasonable degradation representations.
**Algorithm 1:** Training strategy**Input**: A dataset of high quality endoscopic images $X_{h}$, A dataset of low-quality endoscopic videos $X_{l}$, A predefined degradation model PDM   **1****for** *epoch in $0,N_{d}$* **do**  **2**      Sampling $x_{h}$ from $X_{h}$  **3**      Generate ${\hat{x}}_{l}$ by degrading $\mathrm{PDM}(x_{h})$  **4**      Train DAM by minimizing Lcon  **5****end**  **6****for** *epoch in $N_{d},N_{d}+N_{s}$* **do**  **7**      Sampling $x_{h}$ from $X_{h}$  **8**      Sampling $x_{l}$ from $X_{l}$ as images  **9**      Train $G_{L},G_{H}$ by minimizing $L_{\mathrm{adv}}+L_{\mathrm{cyc}}$**10**       Train $D_{L},D_{H},D_{hf}$ by maximizing $L_{\mathrm{adv}}$**11** **end****12** **for** *epoch in $N_{d}+N_{s},N$* **do****13**       Sampling $x_{h}$ from $X_{h}$**14**       Sampling $x_{l}^{t},\ldots ,x_{l}^{t+T}$ from $X_{l}$ as videos**15**       Replace DAM with DRPM**16**       Train DRPM by minimizing $L_{\mathrm{adv}}+L_{\mathrm{cyc}}$**17**       Train $D_{L},D_{H},D_{hf}$ by maximizing $L_{\mathrm{adv}}$**18** **end**

The design of this two-stage dataset utilization scheme is motivated by two key considerations. On one hand, acquiring large-scale paired endoscopic data in clinical practice is extremely challenging, particularly for image pairs exhibiting diverse degradation types, which has a significant impact on the generalization capability of the DAM. On the other hand, models trained solely on artificially degraded pairs often generalize poorly to real surgical videos. By first initializing the degradation representation module with artificial pairs and then adapting the whole model with real unpaired data, our approach combines the advantages of both worlds and ensures better performance in realistic surgical environments.

## 4. Experiments and Results

In this section, we first evaluate the performance of the proposed method on the SCARED dataset [36] and compare it with several state-of-the-art deep learning-based approaches. We then assess the degradation-awareness capability of the proposed module, demonstrating the effectiveness of the learned degradation representations. Furthermore, we evaluate the performance of our method under different degradation patterns across various surgical scenarios. To gain deeper insights into the contribution of each component, we conduct ablation studies. Finally, we construct a simulated surgical environment to examine the real-time performance of the proposed approach.

### 4.1. Data Collection and Implementation Details

#### 4.1.1. Datasets and Preprocessing

In this study, we construct two datasets to support model training and evaluation. For the high-resolution images, we adopt the SCARED dataset. Although originally designed for monocular depth estimation, we select 2295 clear and high-quality left-camera images to build a high-quality endoscopic image dataset. For the low-resolution videos, we utilize the spinal endoscopic surgery dataset (referred to as the SES dataset) collected at Peking University People’s Hospital, from which we extract 240 clips, each consisting of 120 frames. The choice of this dataset is motivated by the fact that Percutaneous Endoscopic Lumbar Discectomy (PELD) is performed in a narrow operative field filled with irrigation fluid, and the image is degraded severely, which may pose different levels of challenge to our method.

Specifically, the resolution of the SCARED dataset is 1280×1024, while the spinal endoscopic dataset has resolutions of 720×1080 or 1920×1080. In order to reduce the redundant areas of the images, such as pure black areas and the inner wall of the working channel, we crop the images into 320×320 patches to construct the low-quality endoscopic dataset. Finally, the SCARED dataset and the SES dataset are randomly split into training, validation, and testing sets following a 7:2:1 ratio, respectively.

#### 4.1.2. Implementation Details

We train our model following the procedure outlined in Algorithm 1. Throughout the training process, we adopt the Adam optimizer with parameters set to $\beta_{1}=0.9$ and $\beta_{2}=0.999$. During the training of the DAM, the learning rate is set to $5\times 10^{-5}$. For the training of the single-frame enhancement model, the learning rates of G and D are set to $5\times 10^{-5}$ and $2\times 10^{-4}$, respectively. For the DRPM, we configure the time interval for DAM-based degradation estimation to 15 frames, with the same learning rate as the previous stage. All experiments are conducted using two NVIDIA RTX 3090Ti GPUs for training, while a single NVIDIA RTX 3090Ti is employed for model testing.

### 4.2. Enhancement Performance and Efficiency of DGGAN

We compare our method with several open-source image enhancement algorithms, including degradation-specific approaches as well as two general-purpose methods (Restormer [37] and SwinIR [38]). For our framework, we evaluate both variants: the degradation-guided enhancement model based on DAM (DGGAN-DAM) and the degradation-guided enhancement model based on DRPM (DGGAN-DRPM).

We first conduct experiments on the SCARED dataset, where the low-quality test images are generated by applying degradations based on multiple degradation models, as defined below. Since the ground truth is available in this setting, we adopt PSNR [39] and SSIM [40] as full-reference metrics to evaluate image quality. We then conduct experiments on the spinal endoscopic dataset, where no ground truth is available. In this case, we employ no-reference metrics, including NIQE [41] and PIQE [42], to assess the performance of different models.

In addition, to evaluate the computational efficiency of each model, we reported two key complexity measures—the number of parameters and the floating point operations (FLOPs)—following common practice in super-resolution studies. To measure the computational efficiency of each model, we calculate two essential properties: the number of parameters and FLOPs (Floating-point operations per second). The number of parameters in a model describes its size, with fewer parameters indicating a more lightweight model. FLOPs can describe the computational complexity of the model and have become a widely used metric to measure the efficiency of models.

#### 4.2.1. Degradation Models

On the SCARED dataset, we apply four degradation types frequently observed in endoscopic surgery: noise, motion blur, low illumination, and electrocautery smoke. For each type, the corresponding degradation model is implemented as summarized in Table 2, and during the training of our DGGAN model, the associated parameters are regressed to support the degradation process.

It should be mentioned that although explicit degradation models and their parameters are calculated, we deliberately choose not to use these parameters as direct supervision signals. Instead, our framework learns to encode and exploit degradation representations that implicitly capture the degradation characteristics. This design enables the model to move beyond parameter-specific regression and achieve greater robustness and generalization across diverse degradations.

For the spinal endoscopy dataset, we further construct a degradation representation model (8) tailored to real surgical scenarios, taking into account four major aspects: low resolution, blurring, low illumination, and underwater imaging effects.

(8) $$I\left(x\right)=\left(\beta {\left(\alpha J\left(x\right)⊗k\right)}^{\gamma }T\left(x\right)+A\left(1-T\left(x\right)\right)\right){↓}_{s}+n$$

The parameter ranges and degradation visual performance are shown in Appendix A.

#### 4.2.2. Results on SCARED Dataset

The performance of multiple models on the SCARED dataset is shown in Table 3 and Figure 6. It is worth noting that since our DGGAN framework incorporates an inherent super-resolution step, for competing methods without an upsampling module, we adopt a strategy of first applying enhancement and then bicubic interpolation for resolution restoration. While this inevitably reduces their final performance, it keeps their inference efficiency comparable.

For the denoising task, we compare against DnCNN [43], CBDNet [44], and Uformer [45]. As denoising represents the simplest enhancement scenario, the performance of all models is remarkable except DnCNN, since it was trained for Grayscale image denoising. Notably, our DGGAN-DRPM achieves results only slightly lower than Restormer and SwinIR, while requiring just 30% and 7.3% of their FLOPs, respectively.

For the deblurring task, we select DeblurGAN [46], MIMOUNet [47], and DBGAN [48]. In this case, DGGAN-DAM achieves the best overall performance, surpassing Restormer by 0.1083 in SSIM, while DGGAN-DRPM reaches 0.7785 SSIM, ranking just behind DGGAN-DAM and SwinIR.

For the low-light enhancement task, we compared against Zero-DCE [49], Retinex-Net [50], and EnlightenGAN [25]. Here, DGGAN-DAM nearly matches Restormer’s performance (a marginal SSIM drop of 0.0038), while DGGAN-DRPM ranks third overall, outperforming SwinIR by 0.0327.

For the electrocautery smoke removal task, we selected DehazeNet [51], FFANet [52], and MSBDN [53]. Again, DGGAN-DAM achieves the best results, though DGGAN-DRPM underperforms compared to Restormer and SwinIR. We attribute this to the inherently stochastic nature of smoke diffusion, which limits the ability of the DRPM to effectively propagate degradation representations across frames.

Overall, these results highlight that even when compared against task-specific enhancement models under comparable efficiency settings, our framework consistently achieves an outstanding balance between computational efficiency and enhancement quality. In particular, for each degradation type, we deliberately choose relatively lightweight baseline models to ensure similar inference efficiency, thereby making the performance gains of our method more directly attributable to its superior balance between efficiency and effectiveness.

In addition, we present the PSNR curves over image frames under DRPM-based degradation representation propagation for four different degradation types, as illustrated in Figure 7. The curves exhibit a downward sawtooth pattern despite the degradation condition remaining unchanged. Specifically, after each DAM-based re-estimation, the restoration quality reaches a local peak. However, as the subsequent degradation representations are obtained through temporal propagation rather than re-estimation, error accumulation gradually occurs, leading to a progressive decline in PSNR. Once the DAM module is triggered again, the degradation representation is re-estimated, and the restoration quality correspondingly improves, forming the observed sawtooth trend.

#### 4.2.3. Results on SES Dataset

We conduct evaluations on our SES dataset subsequently. Although the performance can only be assessed using no-reference quality metrics, specifically NIQE and PIQE, the results in Table 4 and Figure 6 clearly demonstrate that our method significantly outperforms both Restormer and SwinIR. This finding highlights the strong capability of DGGAN in handling complex and diverse degradation scenarios. More importantly, in real surgical environments, the challenges differ markedly from those in general image enhancement tasks. While patient-specific anatomical and physiological conditions may vary, the types of visual degradations that occur during a given minimally invasive surgery tend to remain relatively consistent. For example, in spinal endoscopy surgery, we can systematically characterize the degradation process with a degradation model such as the one formulated in (8). Leveraging this insight, DGGAN is able to achieve reliable and real-time enhancement performance. This implies that, for a specific minimally invasive surgical procedure, it is sufficient to identify and model the major degradation types that are likely to occur. Once this is done, a dedicated enhancement model can be efficiently trained, enabling high-quality and real-time endoscopic video enhancement that directly benefits intraoperative visualization and surgical safety.

#### 4.2.4. Robustness to Abrupt Degradation Change

To evaluate the robustness of the proposed framework under sudden degradation transitions, we design a controlled experiment that simulates abrupt changes in imaging conditions within a continuous video sequence. Specifically, the degradation type and its parameters are intentionally switched at predefined frames, where random motion blur or noise is introduced or removed while low-illumination degradation is consistently present, thereby generating temporally adjacent segments with significantly different degradation characteristics. To enable this evaluation, the model is retrained using data containing combined noise, motion blur and low illumination degradations, ensuring that it can properly handle such composite degradation patterns. All other experimental settings remain identical to those described in the main experiments.

Quantitative and visual results shown in Figure 8 demonstrate that the proposed method maintains stable enhancement performance across most frames despite abrupt degradation shifts. However, a noticeable performance drop is observed in the first few frames immediately after each transition. The output quality in these frames is relatively lower than that in subsequent frames within the same segment, after which the performance rapidly recovers and stabilizes. This behavior indicates that the model possesses a certain degree of robustness to degradation discontinuities but still requires a short adaptation period to fully adjust to newly introduced degradation patterns.

Further analysis reveals that this phenomenon mainly stems from the temporal propagation mechanism employed in the framework. At the moment of degradation change, the degradation representation of the current frame is inherited from preceding frames rather than being instantly re-estimated, which limits the responsiveness of the model to sudden variations. Once sufficient new-frame information is accumulated, the internal representation gradually aligns with the new degradation condition, leading to restored enhancement performance. These observations suggest that while the current design effectively balances efficiency and temporal consistency, incorporating a rapid re-estimation or change-detection mechanism could further improve adaptability to abrupt degradation transitions.

### 4.3. Degradation Representation Performance

Degradation representation learning is a key component of our DGGAN framework, as it enables the model to obtain an implicit estimation of endoscopic image degradations, which in turn guides the overall training process. To validate the effectiveness of this learned representation, we performed a visualization analysis.

Specifically, based on the degradation types defined in Table 2, we apply four levels of artificial degradations to high-quality endoscopic images, denoted as L1–L4. These degraded images are then fed into the DAM to extract their degradation representations. To facilitate interpretation, we further project the high-dimensional representations into a two-dimensional space using PCA [54] for visualization. As shown in Figure 9, the DAM successfully learns meaningful degradation features: different degradation types, such as noise and motion blur, form clearly separable clusters. Even in the case of more complex degradations typical of spinal endoscopic surgery, as modeled in (8), the DAM is still able to capture and distinguish degradation severity to a certain extent, as indicated by the arrow. These results confirm that the DAM provides effective and discriminative degradation representation learning, which forms the basis for robust enhancement performance in diverse surgical scenarios.

### 4.4. Ablation Study

#### 4.4.1. Effectiveness of the Degradation Representation

In this study, we investigate the role of degradation representation in our framework. To this end, we remove the pretraining stage of the DAM module in Algorithm 1 (referred to as DGGAN w/o p), which to some extent simulates the vanishing of degradation representation learning, and compare it with our full model. The visualization results of the learned degradation representations and the corresponding enhancement performance on the SES dataset are shown in Figure 10 and Table 5, respectively. Without pretraining, the model almost completely loses its ability to capture meaningful degradation representations, which directly leads to a significant drop in enhancement performance. We attribute this to the fact that, without pretraining, the DAM is treated merely as a generic feature extractor rather than a degradation-oriented encoder, thus losing its capacity to represent degradation effectively.

#### 4.4.2. Effectiveness of the Degradation Propagation

In this part, we study the impact of different propagation intervals on model performance and inference efficiency. In our default setting, the propagation interval $\Delta_{T}$ is set to 15. To further examine its influence, we test the model with $\Delta_{T}$ values of 3, 5, 10, 20, and 30, and report the average performance and efficiency in Table 6. The results show that smaller $\Delta_{T}$ values yield better enhancement quality but lower inference efficiency, whereas larger $\Delta_{T}$ values achieve the opposite trade-off. This observation is consistent with our intuition: shorter intervals allow more accurate degradation estimation, while longer intervals reduce computational redundancy. Considering that endoscopic videos are typically recorded at 30 fps, we choose $\Delta_{T}=15$ as a balance, which ensures satisfactory enhancement quality while maintaining real-time performance.

#### 4.4.3. Degradation Representation Injection

To validate the design choice of incorporating the degradation representation into the Value branch of the degradation-aware Swin Transformer block, we conduct an ablation study comparing different injection strategies within DGEM. Specifically, we evaluate four alternative conditioning schemes in addition to the used Value modulation mechanism: (1) Query modulation, (2) Key modulation, (3) cross-attention conditioning, and (4) direct feature concatenation. All variants share the same backbone architecture, training data, and optimization settings to ensure fair comparison, differing only in how the degradation representation is utilized.

As listed in Table 7, experimental results on DGGAN-DRPM and its variants show that Value modulation achieves the best trade-off between restoration quality and computational efficiency. Modulating the Query or Key branches leads to slightly inferior PSNR/SSIM performance and introduces higher sensitivity to degradation estimation errors, likely because these branches directly affect attention weight computation, amplifying mismatch effects. Cross-attention conditioning yields competitive enhancement quality; however, it significantly increases computational cost and memory consumption due to the additional attention operations. Direct concatenation exhibits the lowest performance, suggesting that naive feature fusion is insufficient for effectively guiding the attention mechanism.

In contrast, Value modulation preserves the original attention structure while adaptively adjusting feature aggregation according to degradation characteristics. Since only the Value vectors are modulated, the attention weights remain stable, improving robustness to degradation representation noise while maintaining low computational overhead. These results demonstrate that the proposed conditioning strategy offers a balanced solution in terms of enhancement accuracy, stability, and inference speed.

#### 4.4.4. Effectiveness of the Cyclical Consistency

In this study, we analyze the contribution of each component to the cyclical consistency loss. As defined in (7), the cyclical loss consists of three terms, $L_{cl}$, $L_{ch}$, and $L_{cd}$. We conduct ablation experiments by removing each of these terms individually and report the results in Table 8. The results show that $L_{cl}$ and $L_{ch}$ have a major impact on the model’s performance, and removing either of them leads to a substantial drop in enhancement quality. In contrast, the role of $L_{cd}$ is relatively minor, as it mainly penalizes inconsistency in degradation representation. Specifically, $L_{cd}$ improves the stability of degradation estimation and prevents representation drift during propagation. Therefore, $L_{cd}$ serves as a structural regularization term that enhances robustness and theoretical coherence of the degradation modeling framework.

### 4.5. Enhanced Segmentation Performance

To quantitatively evaluate the impact of the proposed video enhancement method on downstream tasks, we conduct vessel segmentation comparative experiments on both the original degraded DRIVE [55] images and the enhanced images. The segmentation network FR-UNet [56] and pretrained model are applied in both settings to ensure a fair comparison, which means the segmentation model is not retrained on our enhanced dataset. As summarized in Table 9, after applying the proposed enhancement algorithm, the segmentation accuracy consistently improves across all evaluation metrics. Specifically, the Area under Curve (AUC) increases from 0.9517% to 0.9582%, while the Intersection-over-Union (IoU) improves by 2 percentage points.

Qualitative results in Figure 11 further demonstrate that the enhanced images exhibit clearer tissue boundaries, improved local contrast, and reduced artifacts, which effectively facilitate the segmentation network’s discrimination between adjacent anatomical regions. These results validate that the proposed image enhancement algorithm not only improves visual quality but also provides a more reliable and discriminative input for tissue segmentation. This confirms the practical value of the enhancement module as a preprocessing step for downstream clinical perception tasks, particularly in challenging endoscopic surgical scenarios.

## 5. Conclusions

We developed and validated an efficient stepwise endoscopic video enhancement framework. Our approach estimates degradation representations of images and propagates them across video frames, thereby enabling high-quality real-time enhancement of endoscopic videos. By achieving a superior balance between performance and efficiency compared with several state-of-the-art methods, our work demonstrates the value of degradation representations in image enhancement. Moreover, this degradation-guided enhancement paradigm implies that, for endoscopic images in a specific minimally invasive surgery, it is sufficient to evaluate and model the potential degradations to train an enhancement model, which can then deliver high-quality real-time enhancement during surgery.

Nevertheless, our method has certain limitations. On the one hand, to ensure real-time performance, the parameter size of the single-frame enhancement module was reduced, leading to a performance gap compared with the best-performing state-of-the-art methods. On the other hand, our current design employs a fixed interval for degradation feature estimation, which may limit optimal performance. For instance, sudden events such as tissue bleeding may not be captured promptly, resulting in suboptimal enhancement outcomes due to delayed degradation estimation.

Future research will concentrate on two aspects. The first is comprehensive end-to-end evaluation in real surgical settings to verify the practical feasibility of real-time application. The second involves leveraging the emerging capability of large language models (LLMs) for medical image analysis, as evidenced by prior studies [57,58], to incorporate LLM-guided or vision-language reasoning for high-level scene interpretation, adaptive key-frame scheduling, and controllable enhancement through surgeons’ natural-language constraints. These directions aim to enhance clinical reliability and accelerate real-world deployment.

## Figures and Tables

**Figure 1:**
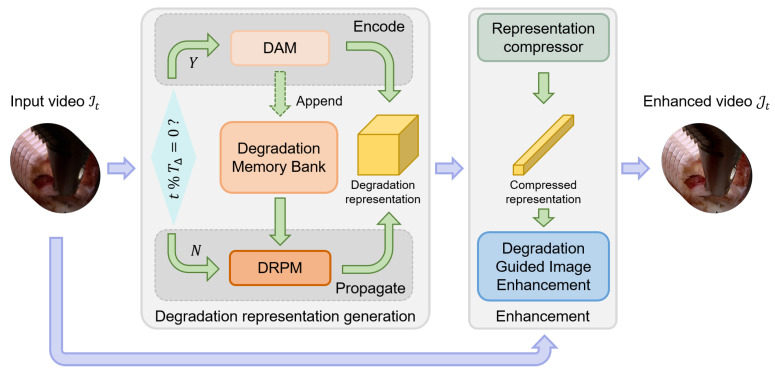
Framework of the real-time video enhancement. Each frame of the input video sequence undergoes degradation representation modeling and generative enhancement. Specifically, for frames whose index is a multiple of $T_{\Delta }$, the system invokes the degradation-aware module (DAM) to perform a full estimation, yielding high-precision degradation features. For frames that are not multiples of $T_{\Delta }$, the degradation representation is rapidly propagated through the degradation representation propagation module (DRPM) along the temporal dimension and subsequently fed into the single frame enhancement model to enhance the current frame. All the modules are detailed in the following sections.

**Figure 2:**
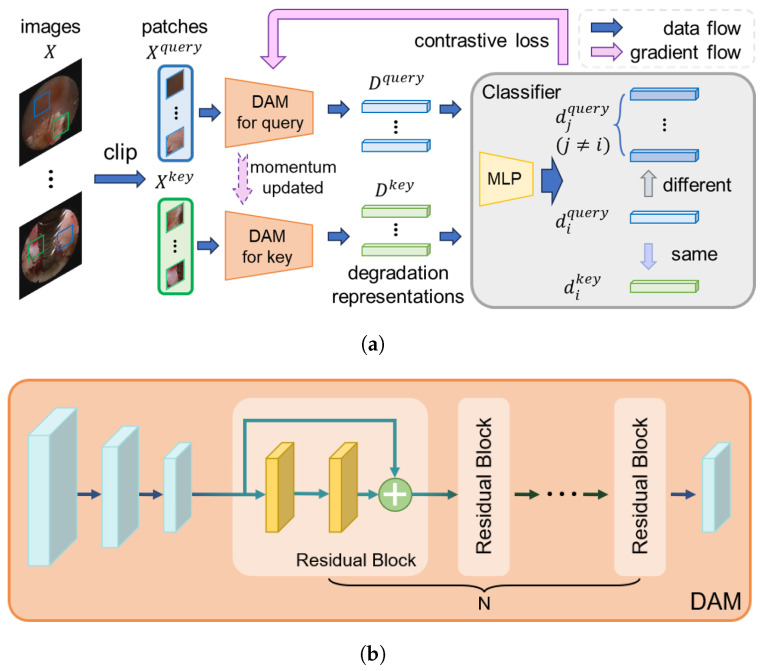
The diagram of the DAM. (**a**) The DAM training workflow. (**b**) The structure of the DAM.

**Figure 3:**
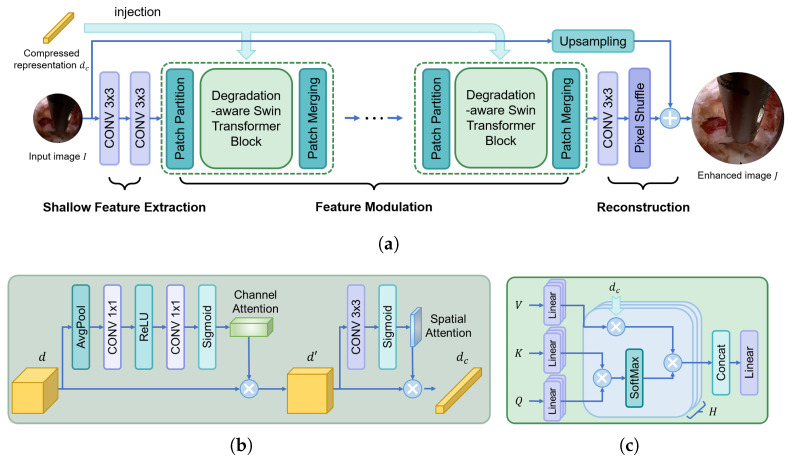
Degradation guided enhancement module (DGEM). (**a**) The basic architecture of the DGEM, which includes four parts: Degradation representation compression, shallow feature extraction, feature modulation, and Reconstruction. (**b**) The degradation compression block, which implements both channel attention and spatial attention mechanisms. (**c**) The Degradation-aware Swin Transformer Block, which injects the degradation representation $d_{c}$ into the value component and modulates the input feature based on multi-head self-attention.

**Figure 4:**
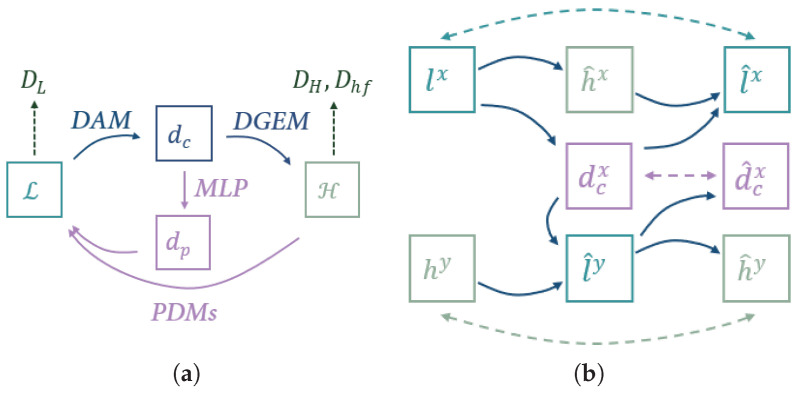
Diagram of the cyclical consistency. (**a**) The dataflow of the whole model. L→H: achieved by the DAM and the DGEM with an additional output $d_{c}$. H→L: achieved based on PDMs and the regression of $d_{p}$. (**b**) Calculation of the cyclical consistency loss. Solid lines indicate the dataflow when generating reconstructed synthetic images, and dashed lines indicate the cyclical consistency loss calculation.

**Figure 5:**
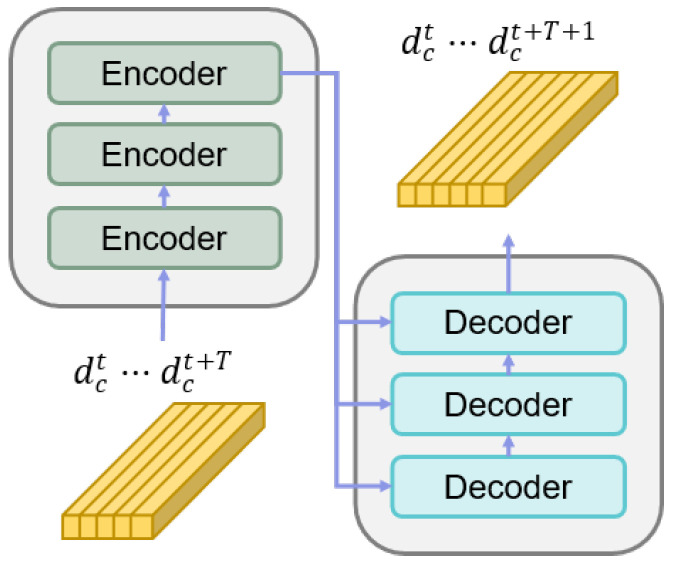
The architecture of the DRPM.

**Figure 6:**
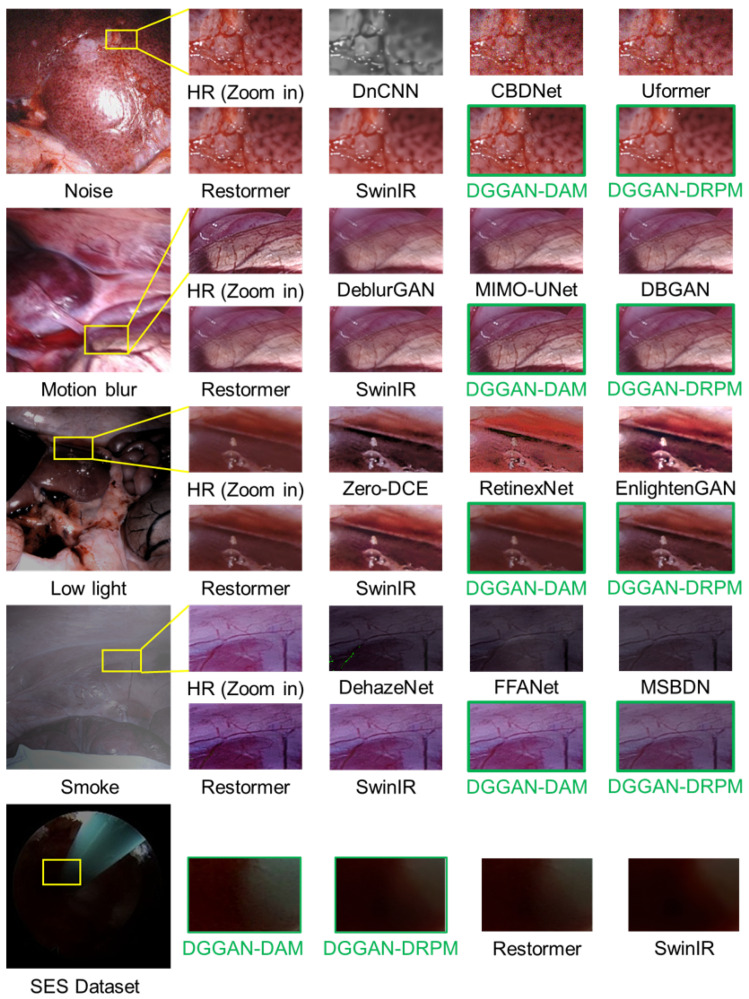
Qualitative comparison of enhancement results on degraded endoscopic datasets. The first four rows correspond to the SCARED dataset under four degradation types: noise, motion blur, smoke, and low light. The last row shows results on our SES dataset.

**Figure 7:**
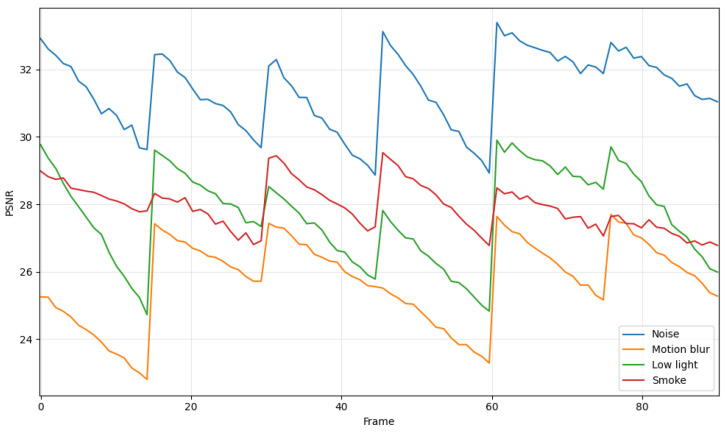
The PSNR curves over image frames under DRPM-based degradation representation propagation for four different degradation types.

**Figure 8:**
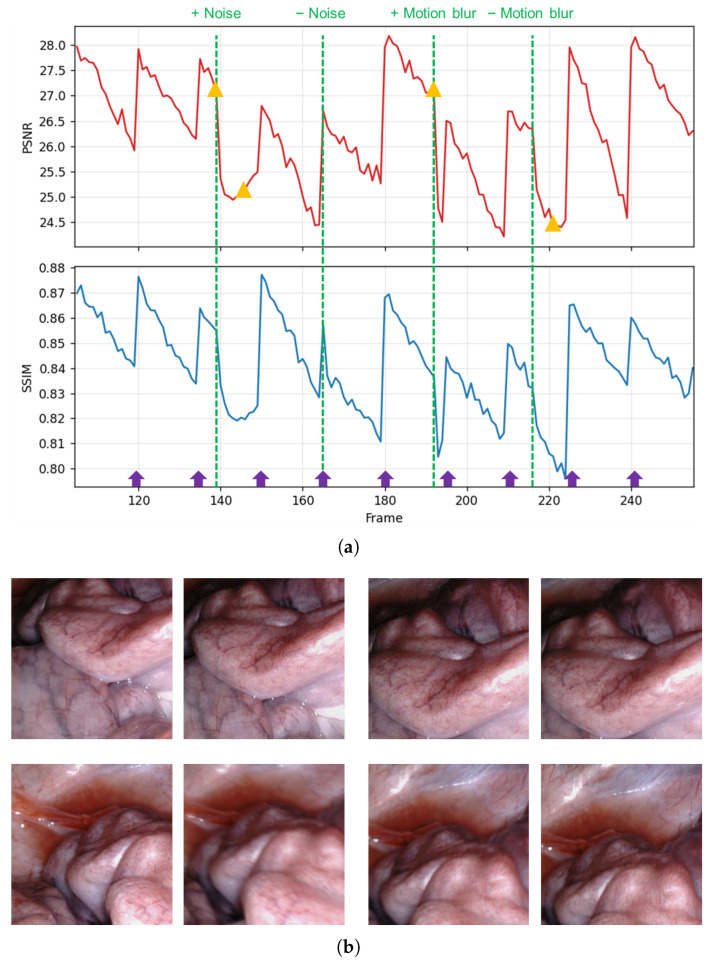
Performance when abrupt degradation change. (**a**) Temporal variation of PSNR and SSIM over time. The purple arrows indicate frames in which the degradation representation is estimated by the DAM module, while the green line denotes the timing and pattern of degradation transitions. (**b**) Visual enhancement performance of frames before and after the time points marked by the yellow triangles in (**a**).

**Figure 9:**
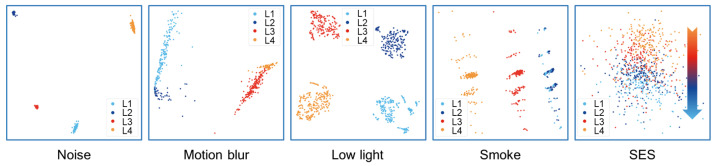
The degradation representation performance of the DAM under different degradation types. Arrows indicate the distribution of representations under different degradation levels.

**Figure 10:**
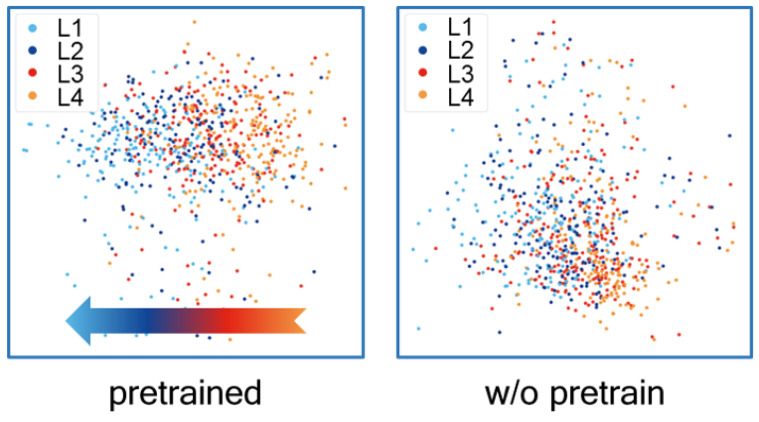
The degradation representation performance on SES dataset with or without pretraining. Arrows indicate the distribution of representations under different degradation levels.

**Figure 11:**
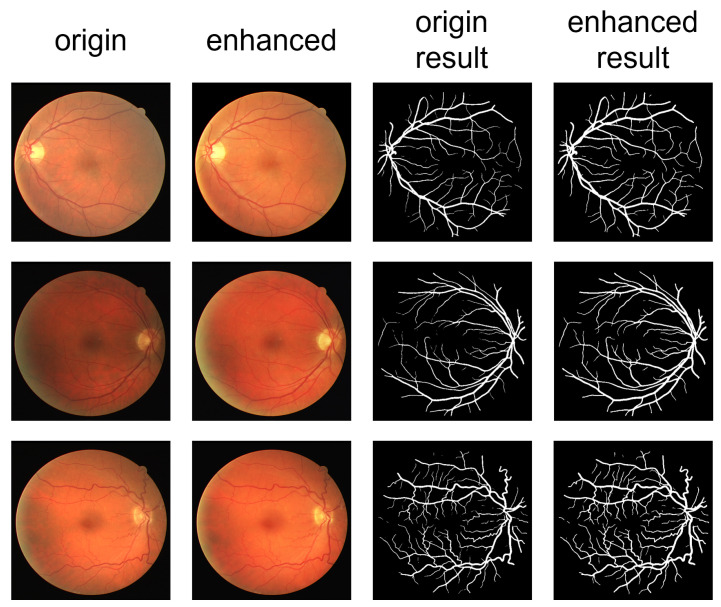
The enhanced performance on the DRIVE dataset and the downstream segmentation performance comparison.

**Table 1:** Number of parameters and FLOPs for each part of DGGAN.

	Params (M)	GFLOPs
DAM	4.33	29.75
DGEM	0.39	24.65
Degradation Representation Compression	0.066	1.31
Shallow Feature Extraction	0.0018	0.11
Feature Modulation	0.13	8.22
Reconstruction	0.19	15.01

**Table 2:** The corresponding models for different degradation types.

Degrade Type	Degrade Model
Random Noise	I(x)=J(x)+n(x)
Motion Blur	I(x)=k(x)⊗J(x)
Low Light	I(x)=J(x)·L(x)+n(x)
Smoke	I(x)=J(x)t(x)+A(1−t(x))
SES	referred as to (8)

**Table 3:** Performance comparison in terms of PSNR (dB), SSIM, parameter counts, FLOPs, and running time (s) of the proposed DGGAN with the SOTAs on the SCARED dataset. Bold indicates the best result, underline indicates the second-best result. ↑ and ↓ denote whether a higher or lower value represents better performance, respectively.

Degradation	Method	PSNR (dB) ↑	SSIM ↑	Parameters (M) ↓	GFLOPs ↓	Running Time (s) ↓
Random Noise	DnCNN	15.50	0.7265	0.56	28.11	0.0205
	CBDNet	28.98	0.8185	4.43	30.84	0.0385
	Uformer	30.96	0.8534	20.63	41.09	0.0419
	Restormer	31.52	0.8710	26.13	108.0	0.5271
	SwinIR	32.76	0.8902	10.89	711.9	11.242
	DGGAN-DAM	**33.21**	**0.9057**	4.72	54.41	0.1459
	DGGAN-DRPM	31.03	0.8678	0.63	32.40	0.0291
Motion Blur	DeblurGAN	12.87	0.4879	6.06	26.88	0.0738
	MIMOUNet	17.59	0.6783	6.81	51.26	0.0083
	DBGAN	18.54	0.7121	11.59	74.35	0.1301
	Restormer	23.01	0.8127	26.13	108.0	0.6159
	SwinIR	22.97	0.8094	10.89	711.9	10.855
	DGGAN-DAM	**26.86**	**0.9210**	4.72	54.41	0.1421
	DGGAN-DRPM	20.85	0.7785	0.63	32.40	0.0305
Low Light	Zero-DCE	19.11	0.7085	0.024	1.178	0.0018
	RetinexNet	19.16	0.7838	0.56	16.78	2.4734
	EnlightenGAN	19.26	0.7755	54.41	127.45	0.1187
	Restormer	**28.83**	**0.8657**	26.13	108.0	0.5764
	SwinIR	25.49	0.8018	10.89	711.9	12.843
	DGGAN-DAM	28.01	0.8619	4.72	54.41	0.1399
	DGGAN-DRPM	26.54	0.8345	0.63	32.40	0.0314
Smoke	DehazeNet	16.86	0.6899	0.02	0.391	0.5237
	FFANet	23.05	0.7311	4.68	231.2	0.3891
	MSBDN	19.01	0.6529	28.71	18.81	0.1031
	Restormer	27.37	0.8346	26.13	108.0	0.6038
	SwinIR	26.79	0.7981	10.89	711.9	11.745
	DGGAN-DAM	**28.18**	**0.8553**	4.72	54.41	0.1407
	DGGAN-DRPM	27.13	0.7823	0.63	32.40	0.0276

**Table 4:** The Evaluation results of NIQE/PIQE on our SES dataset. Bold indicates the best result, underline indicates the second-best result. ↓ denote a lower value.

	Method	NIQE ↓	PIQE ↓	Parameters (M) ↓
SES	Restormer	4.32	15.79	26.13
	SwinIR	5.03	18.21	10.89
	DGGAN-DAM	**3.62**	**13.79**	4.72
	DGGAN-DRPM	3.98	16.82	0.63

**Table 5:** The enhancement performance with or without the DAM pretraining. Bold indicates the best result. ↑ and ↓ denote whether a higher or lower value represents better performance, respectively.

Setup	SCARED-Noise		SES	
	PSNR↑	SSIM↑	NIQE↓	PIQE↓
DGGAN w/o p	27.21	0.8552	6.19	22.87
DGGAN	**33.21**	**0.9057**	**3.62**	**13.79**

**Table 6:** The enhancement performance under different propagation interval $\Delta_{T}$. Bold indicates the best result. ↑ and ↓ denote whether a higher or lower value represents better performance, respectively.

$\Delta_{T}$	SCARED-Noise		SES		Running Time (s)
	PSNR↑	SSIM↑	NIQE↓	PIQE↓	
3	**32.54**	**0.8844**	**3.64**	**14.06**	0.0973
5	32.18	0.8803	3.70	14.49	0.0525
10	31.79	0.8776	3.77	15.13	0.0408
15	31.03	0.8678	3.98	16.82	0.0369
20	30.18	0.8595	4.09	17.92	0.0350
30	28.32	0.8391	4.27	19.03	**0.0330**

**Table 7:** The enhancement performance using different degradation representation injection. Bold indicates the best result. ↑ and ↓ denote whether a higher or lower value represents better performance, respectively.

Setup	SCARED-Noise		SES		Running Time (s)
	PSNR↑	SSIM↑	NIQE↓	PIQE↓	
query modulation	28.71	0.8483	4.76	18.03	0.0293
key modulation	29.46	0.8552	4.14	16.99	0.0290
cross attention	**31.97**	**0.8893**	**3.69**	**16.45**	0.0312
feature concatenation	24.83	0.7764	7.63	23.91	**0.0288**
Ours	31.03	0.8687	3.98	16.82	0.0291

**Table 8:** The effectiveness of the cyclical consistency loss. Bold indicates the best result. ↑ and ↓ denote whether a higher or lower value represents better performance, respectively.

Setup	SCARED-Noise		SES	
	PSNR↑	SSIM↑	NIQE↓	PIQE↓
w/o $L_{cl}$	21.53	0.5210	9.38	22.81
w/o $L_{ch}$	18.87	0.4983	11.01	25.75
w/o $L_{cd}$	32.77	0.8842	4.03	15.80
Ours	**33.21**	**0.9057**	**3.62**	**13.79**

**Table 9:** The vessel segmentation performance on both the origin and the enhanced DRIVE dataset using FR-UNet. Bold indicates the best result.

	AUC	F1 Score	Accuracy	IoU
Original	0.9517	0.8092	0.9639	0.6798
Enhanced	**0.9582**	**0.8133**	**0.9671**	**0.6994**

**Figure A1:**
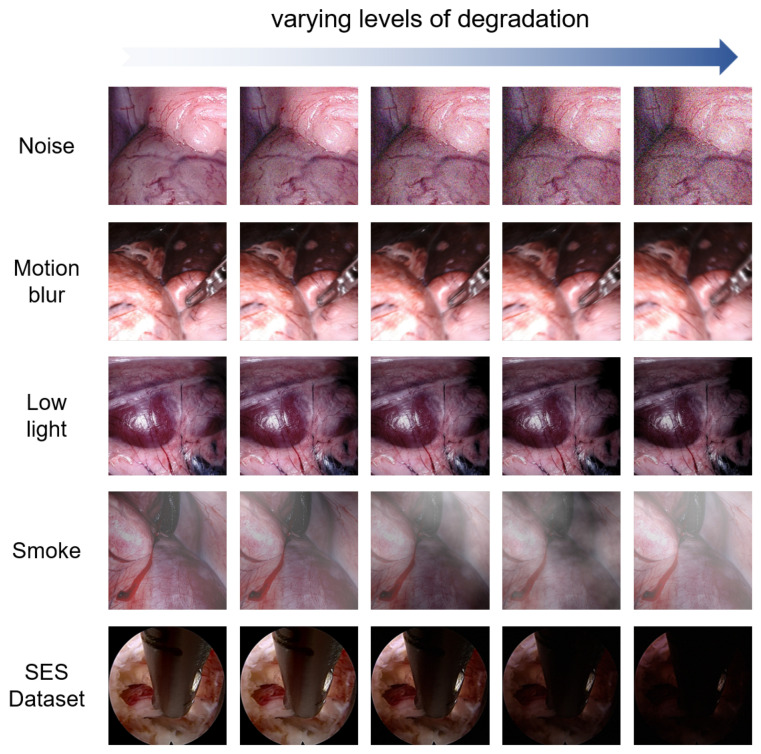
The endoscopic images under varying degradation levels.

## Data Availability

The data presented in this study are openly available in SCARED at https://endovissub2019-scared.grand-challenge.org/ (accessed on 3 May 2024).

## Appendix A

This appendix summarizes the parameter ranges for each degradation type discussed in the manuscript and illustrate the endoscopic images under varying degradation levels in Figure A1.

Random noise: Random Gaussian noise with intensity in the range of [0, 30].

Motion blur: Random Gaussian blur where the kernel size k∈[3,19], the standard deviation σ∈[0.2,4.0], and the motion angle $\theta ∼U\;(-45^{\circ },45^{\circ })$.

Low light: The image is converted to HSV, where the normalized V channel is additively modulated within [−0.25, 0], then converted back to RGB with random noise in [0, 10].

Smoke: Fog is synthesized by adding a plasma-fractal haze field [59] with intensity α∈[1.0,3.0] and roughness β∈[1.4,2.0], followed by contrast compression and clipping.

SES: The size of the isotropic Gaussian kernel *k* in (8) is fixed to 7×7, and the range of the width of the kernel σ is set to [0.2, 4.0]. The noise level range *n* is set to [0, 30]. Then, the parameters α,β,γ are set in range of [0.9, 1.0], [0.8, 1.0] and [1.0, 2.5], respectively. The attenuation coefficients are set to $\beta_{R}\in \left[1.5,3.0\right],\beta_{G}\in \left[0.8,1.5\right],\beta_{B}\in \left[0.3,0.8\right]$ with $\beta_{R}>\beta_{G}>\beta_{B}$. The background light B∈[0.05,0.8], and the depth is normalized to [0, 1].

As illustrated in Figure 4, these parameters can cover the range of image degradation in PELD endoscope scenarios.
